# A qualitative analysis of suicidal psychiatric inpatients views and expectations of psychological therapy to counter suicidal thoughts, acts and deaths

**DOI:** 10.1186/s12888-018-1921-6

**Published:** 2018-10-16

**Authors:** Yvonne F Awenat, Sarah Peters, Patricia A Gooding, Daniel Pratt, Emma Shaw-Núñez, Kamelia Harris, Gillian Haddock

**Affiliations:** 1 Division of Psychology and Mental Health, School of Health Sciences, University of Manchester, Zochonis Building, Brunswick St, Manchester, M13 9PL UK; 2 Manchester Academic Health Science Centre, MAHSC, Manchester, UK; 3 Manchester Centre for Health Psychology, University of Manchester, Manchester, UK; 4 Greater Manchester Mental Health NHS Trust, Manchester, UK

**Keywords:** Suicide prevention, Psychiatric inpatients, Psychological therapy, Qualitative, User-views, Implementation

## Abstract

**Background:**

Suicide is a global problem and suicidal behavior is common in acute psychiatric wards. Inpatient suicides regularly occur with 10.4/100,000 such deaths recorded in the UK in 2016. Inpatient suicides are potentially the most avoidable of all suicides as inpatients have 24-h staff contact. Current inpatient treatment prioritizes maintenance of physical safety by observation, medication and general supportive measures, however efficacious and effective specific treatments are lacking. Psychological treatments have a growing evidence base for suicide prevention yet provision of inpatient therapy is uncommon. The present qualitative study aimed to understand the patient acceptability issues by investigating suicidal inpatients views and expectations of a novel suicide-focussed cognitive behavioural psychological therapy which was nested alongside a pilot clinical trial of the intervention.

**Methods:**

Thematic analysis of semi-structured individual qualitative interviews with twenty suicidal psychiatric inpatients to investigate their views and expectations about ward-based suicide-focused psychological treatment.

**Results:**

Two main themes were identified. The first, ‘A therapy that works’, revealed inpatients’ views of the necessary components for effective ward-based suicide-focused psychological therapy. The second, ‘Concerns about in-patient suicide-focused therapy’, depicted their fears about engaging in this treatment. Results suggested that suicide-focused psychological therapy was cautiously welcomed by inpatients’ whose narratives expressed their needs, priorities and concerns. Further data analysis enabled formation of a user-informed model of suicide-focussed psychological therapy which offers guidance for researchers and clinicians.

**Conclusions:**

We conclude that hospitalization of suicidal individuals offers a critical opportunity to intervene with effective treatment to preserve life and that suicide-focussed psychological therapy is likely to be well received by suicidal inpatients warranting further testing with a sufficiently powered definitive trial. It is important that provision of ward-based psychological therapy for suicidal inpatients addresses the considerable context-specific challenges inherent in this setting.

**Trial registration number:**

ISRCTN 17890126, Registry: UK Clinical Trials Gateway, Date of registration: 22/04/15, Date of enrolment of first participant to the trial: 20/05/14 (retrospectively registered).

## Background

Suicide is a global problem equating to one death and 20 attempted suicides every 40 s [1]. Internationally suicide rates vary, with 13.4/100,000 population suicide deaths recorded for 2016 in USA [2] and 10.91/100,000 population suicides recorded across Europe [3] during 2015. UK data for 2016 indicates 13.4/100,000 suicide deaths of which 28% were by mental health patients, 9% of which are attributed to inpatients [4]. Morbidity from repetitious suicidal behaviour is common in UK psychiatric wards where individuals at high risk of suicide are frequently admitted [5]. Psychiatric in-patient treatment prioritizes maintenance of physical safety by observation, medication and general supportive measures, yet despite 24-h staff presence, in-patient suicides persist, with 1443 suicides recorded during 2005–2015 [4, 5].

International health reforms driven by greater focus on community based treatment and economic pressures have resulted in reduced psychiatric bed availability in the USA [6], Europe [7], Israel [8] and in the UK, where 42% of psychiatric beds were closed during 2000–2012 [9]. Consequently, pressure on remaining beds has resulted in shorter hospitalization episodes, with those lasting less than one week being linked to post-discharge suicide [4]. In-patient suicides are particularly preventable as the 24-h staff contact offers a unique opportunity to intervene [10], yet effective suicide prevention interventions for in-patients remain elusive.

Psychological therapy has a growing evidence base for suicide prevention, for example: dialectical behavioural therapy (DBT) [11], psychodynamic-interpersonal therapy [12], suicide-focused counselling [13], and cognitive behaviour therapy (CBT) [14–18]. A systematic review and meta-analysis [18] of several forms of psychological therapy found CBT to demonstrate superior outcomes for repetition of self-harm at 12 months follow-up (OR 0.80, 95%; CI 0.65–0.98; 10 trials; *n* = 2232), which accords with the results of an earlier systematic review [14] which also found CBT to be most efficacious. The UK National Institute for Health and Care Excellence (NICE) [16]. recommends CBT for suicidal patients in any treatment setting. However, despite international recognition of the value of psychological treatment for suicidal in-patients (e.g., South Africa [19], USA [20]; UK [16], Sri Lanka [21], India [22]), provision remains inconsistent. Within UK psychiatric hospitals suicidal in-patients rarely receive psychological therapy as usual practice favours post-discharge referral for psychological treatment [23]. Consequently, publications of studies about adult in-patients’ experiences of psychological suicide-prevention treatments are scarce. Several studies of community-dwelling patients’ evaluations of suicide-focused psychological interventions exist [24–26], all reporting generally positive experiences and satisfaction of participants. Most participants continued to experience suicidal ideation but perceived that therapy had equipped them with skills to avert transition of thoughts to suicidal behaviour [24–26]. However congruence of views and experiences of community and in-patient populations cannot be assumed given their different clinical and contextual situations [27].

Evaluations of psychological treatments for suicide-prevention commonly report standardised measures of psychopathology, which although crucial to quantifying clinical efficacy, fail to address patient expectations and experiences which, as determinants of treatment response, also impact on clinical efficacy [28]. It is therefore important that therapy is ‘efficacious’ in terms of having demonstrated positive clinical outcomes for research participants under strictly controlled scientific conditions, *and* is ‘effective’ in terms of real-world patient acceptability [29]. Where patient experience of suicide-focussed therapy has been studied, the focus is typically on post-therapy evaluations [24–26], however, this fails to elicit the beliefs and expectations of *potential* recipients [28]. Patient’s ‘expectancy effects’ [30] and role expectations of self and therapist influence the quality of the therapeutic alliance [31], which, as a component of efficacy [32], is essential for therapeutic change [33, 34]. Such constructs are suggested to account for 15–33% of the treatment effect [30, 35], indicating the importance of attention to such issues during treatment innovation as recommended by the UK Medical Research Council [36].

As uptake of and adherence to psychological interventions by suicidal populations is problematic [37–39], it is imperative to develop therapies that are acceptable to patients. Within health behaviour theory [40] Janz’s value-expectancy health belief model [41] offers guidance for improving adherence to psychological treatments by suicidal patients, purporting that an individual’s willingness to enact change behaviour (i.e., uptake psychological therapy) is dependent on how likely that action is perceived to achieve their desired outcome. It is therefore important that patients comprehend and have confidence in their treatment which accords with UK National Health Service (NHS) priorities [42].

Historical literature portrays the challenges and complex interpersonal dynamics for suicidal inpatients and professional staff alike with Morgan’s [43] conceptualisation of ‘malignant alienation’ whereby pernicious contextual psychosocial forces within inpatient wards alienate staff from inpatients and vice versa. Indeed, contemporary qualitative accounts of inpatients’ experiences of psychiatric hospitalisation continue to attest to perceptions of un-therapeutic regimes which fail to meet their psychological needs [19, 44, 45]. Whilst there is an urgent need for effective treatments for suicidal in-patients whose hospitalisation offers a critical opportunity to avert suicide, the psychiatric ward setting itself presents major contextual challenges [46], including the potential of exacerbation of self-harm behaviour [47] and heightened suicide risk [48]. It is important that treatments aimed at helping are acceptable to the suicidal patients as negative experiences of suicide-prevention treatments may worsen the patients’ condition [49]. The qualitative study presented in this paper therefore aimed to investigate suicidal in-patients’ views and expectations of a novel ward-based suicide-focussed psychological therapy intervention nested within a pilot feasibility clinical trial [50]. We believe this to be the first study of this population and topic.

## Methods

### Design

This qualitative study was nested within a mixed-method feasibility study involving a pilot clinical trial which was conducted from October 2013 to December 2016 across five acute adult psychiatric wards of a NHS mental health service in Northern England. Qualitative approaches have been successfully applied in previous studies of suicidal patients views [18, 25, 49, 51] and were therefore selected as suitable to investigate the plethora of ‘unknowns’ regarding potential therapy recipients’ views, perceptions and expectations of a new suicide-focussed psychological treatment. Data collection was by face-to-face semi-structured individual interviews to provide inpatient participants’ with a calm and private setting conducive to discussion of personal and potentially sensitive views [51].

### Inclusion and exclusion criteria

Participants were current in-patients of a public mental health hospital in Northern England who were aged 18 years or over and had self-reported experience of suicidal thoughts or behaviour within the past three months. Participants were excluded if they lacked capacity to provide informed consent.

### Recruitment

Ethical approval was granted by a statutory UK Research Ethics Committee (13/NW/0504). Recruitment for this qualitative study occurred during the consultation period during which trial procedures and therapy content were being refined [50], hence participants had real potential to influence the intervention. Purposive sampling [52, 53] was employed to recruit current inpatients who met the eligibility criteria necessary to provide data to address the aims of this research. All in-patients were informed about the study by ward staff in the first instance. Those who expressed interest in taking part were then contacted by either the first and fifth authors who provided further verbal and written information and allowed a minimum of 24 h for individuals to consider their decision. All patients informed about the study (*N* = 20) expressed interest and subsequently provided written informed consent. Researchers involved in direct contact with participants were trained in ethical participant care including assessment of participant’s capacity to provide informed consent. An ‘ethics-as-process’ approach [54] was taken whereby assessment of capacity was conducted during inpatients’ initial meeting with researchers and then re-confirmed during the subsequent meeting prior to obtaining formal written informed consent, and again prior to and during qualitative interviewing. Recruitment continued until theoretical saturation was reached at which point sufficient data to satisfy the study aims had been collected [52].

### Semi-structured interview

Given the sensitive topic and participants’ vulnerability, considerable attention was invested in interviewer training to ensure participant safety [51]. A semi-structured interview schedule based on current literature with further refinement and piloting by a Service User Group (comprised of eight people with past experience of psychiatric inpatient treatment for suicidality) was developed. Interview questions were ‘funnelled’ to present opening questions about general experiences of ward treatments and psychological therapy prior to suicide-focused questions (e.g., views about suicide-focussed psychological therapy), finally ending with less personal topics (e.g., views of the interview process). Interviews were digitally audio-recorded and transcribed verbatim (at which point all identifying data was anonymised) following which recordings were deleted. All participants were debriefed post-interview to ensure provision of appropriate support for any distress.

### Analysis

Analysis was led by the first author assisted by the second, fifth and sixth authors who contributed to initial and iterations of coding with further contributions to critical discussions of analytic interpretations from the third, fourth and seventh authors thereby enhancing trustworthiness of the final themes [55]. Inductive Thematic Analysis, recognised as a systematic method of identifying thematic patterns across the data corpus was employed utilizing Braun and Clark’s [56] recommended six-phase analytical procedures. We adopted constant comparative approach whereby data generation and coding occurred in parallel to enable ongoing analysis to inform questioning in subsequent interviews [56, 57]. This was achieved by reflection on field notes taken during interviews and review of recent audio-recordings to identify topics requiring further probing or participant generated issues not listed in the core interview schedule. As such, this represented the first phase of analysis although not being a discreet entity, this was continued throughout the analytic process. First phase familiarisation with the data continued by reading each transcript several times to identify potential relevant areas of interest. Moving into the second phase of analysis involved manual line-by-line analysis to name segments of data as tentative codes, following which in the third phase codes with a common meaning were clustered together as potential themes. During the fourth and fifth phases theme names and contents were reviewed and subjected to critical interrogation by the wider research team to check their data-driven genesis. The final sixth phase comprised preparation of the manuscript involving conceptualising the explanatory narrative and selection of the most pertinent data extracts as illustrative quotes.

A reflexive stance of ‘empathic neutrality’ was taken by the research team who recognised the potential influence of personal beliefs and preconceptions on analytic processes yet aspired to interpret and report participants’ narratives as true to their experience. The research team comprised academics (a doctoral student and junior / senior researchers) and academic / clinicians (two Clinical Psychologists’ and one Registered Nurse) assisted by the Service User Reference Group comprised of eight individuals’ with prior experience of psychiatric hospitalisation and suicidality. This enabled a wide range of views to be considered during research meetings where analytic interpretations were discussed.

## Results

### Participants

Twenty participants, comprising fourteen males, were recruited from five acute psychiatric wards in a NHS psychiatric hospital in Northern England, UK. Self-reported demographic data indicated participants’ ages ranged from 22 to 65 years (median 38.1); duration of hospitalisation spanned from two to 672 days (median:21); and previous admissions during the past year ranged from none to three. Participants self-reported their reasons for admission, choosing to provide their diagnosis or perception of a precipitant event. Further details are provided in Table 1.

**Table 1:** Participant Socio-demographic Information

Ppt.	Reason for admission	Length of stay on ward in days	Admissions in last 12 months	Diagnosis	Previous psychological therapy
1.	Hearing voices	21	1	SCZ^4^	No
2.	Overdose	21	1	U	Yes
3.	U	112	u	MDD^2^	No
4.	Sectioned	336	u	Alcoholism	No
5.	Breakdown; overdose	10	2	U	Yes
6.	Repeated overdoses	49	1	Emotionally unstable PD^1^	No
7.	Overdose (Sectioned)	u	u	Anxiety	Yes
8.	U	u	u	U	No
9.	Manic / high; overdose	38	u	Bipolar / PD^1^	Yes
10.	Hearing voices	21	1	Voices / SCZ^4^	No
11.	Suicidal	17	1	U	No
12.	U	14	1	Bipolar / PD^1^	No
13.	U	84	2	SCZ^4^	No
14.	Suicidal	21	0	Depression	No
15.	Stopped clozapine	7	0	SCZ^4^	No
16.	Suicidal	10	0	U	No
17.	Self-harm	672	u	PTSD^2^ / Emotionally unstable PD^1^	No
18.	Feeling down	2	0	Depression	No
19.	Mental breakdown	7	0	U	No
20.	Hearing voices	12	3	U	No

*Abbreviations***:** Gender *M* Male, *F* Female, *PD*^*1*^ Personality Disorder; P*TSD*^*2*^ Post Traumatic Stress Disorder, *MDD* Major Depression Disorder^3^, *SCZ* Schizophrenia ^4^, *U* data unavailable

### Overview of key findings

Analysis identified two themes (Table 2), each with three sub-themes. Theme 1 - ‘A therapy that works’, epitomised participants’ perceptions of the influences and necessary components for effective ward-based suicide-focused psychological therapy. Theme 2 - ‘Concerns about in-patient suicide-focused therapy’ depicted participants’ fears about engaging with therapy. Findings were further synthesized to develop a user-informed model (Table 3).

**Table 2:** Themes and Sub-themes

Theme 1: A Therapy that ‘works’	Theme 2: Concerns about inpatient suicide -focused therapy
Past experiences shaped expectations	A secure therapeutic relationship
Suicidality-specific goals	Potential for harm
Mechanisms: how suicide-focussed therapy works	Ending therapy

**Table 3:** User-informed conceptual model of in-patient suicide-focused psychological therapy [52]

Stage of therapy	Client’s Need	Supporting data	Therapeutic approach
Immediate	Feel safe. Overcome fear of talking about suicide.	*“… it might make me more suicidal. Because the question is being asked all the time and even though I have tried to take my life numerous times …And if it’s prevalent, if it’s there and somebody’s reminding you of it, you’re more likely to do it” (P02)*	Explore potential barriers to therapy and if necessary defuse fears of talking about suicide.
	Development of strong therapeutic relationship.	*“You can go and just relax and like express yourself with that person, with free of them judging you*. *.*.” *(P19)*	Create a safe environment conducive to building secure, ‘containing’ therapeutic relationship. Promote trust by empathic validation of client’s distress and by allowing client to set the pace and depth of discussions. Demonstrate collaboration with client by negotiating acceptable levels of information sharing with ward staff.
	Catharsis / Relief from distress.	*“Being able to talk about what you’ve done [suicidal behaviour] and someone to listen” (P06)*	Facilitate client to share experiences of suicidal ideation and behaviour by demonstrating non-judgement and empathy. Normalise client’s experiences to promote a sense of feeling understood. Self-soothing by relaxation/breathing practices. End of session grounding techniques during last 5–10 min.
	Tolerating intense negative emotions / suicidal thoughts and prevention of suicidal behaviour.	*“I’d be worried what will happen once that barrier’s been broke down to tell you the truth. Because I don’t know whether I’d start crying or get angry.” (P14)*	Guide development and practice of distress tolerance skills / techniques to overcome emotional avoidance and emotional dysregulation. Develop attentional control, attentional broadening and switching techniques to reduce threat-based information processing biases. Promote clients’ sense of agency by assisting recall of experiences of overcoming suicidal states.
Intermediate	Make sense of suicidal thoughts and behaviour.	*“Trying to get rid of the suicidal thoughts, just talking about the suicidal thoughts… and why I’ve got them… talking about it would just help really ‘cos there’s no one to talk to about it, so it’d be best to just to have someone to talk to about the suicidal thoughts and what they’re about.” (P10)*	Collaborative development of individualised formulation. Foster therapist - client’s shared understanding of drivers and inhibitors of suicidality/suicidal behaviour. Identify therapeutic goals targeting suicide reduction. Reflect on experiences of helpful and unhelpful escapes from distress.
	Self-understanding and self-management of emotions and cognitions.	*“Help me to recognise when I’m going to be suicidal and perhaps be able to do something about it.” (P02)*	Provide exit points from suicidal thoughts and cognitions by: - Identifying and challenging negative self-appraisals. - Cultivating emotional regulation skills and positive affect / self-image techniques. - Generating problem-solving strategies to manage threats associated with suicidal cognitions.
	Regain personal independence, and social confidence / functioning.	*“It’s more like building up my social skills a bit more and like talking to people in a group”. (P19)*	Behavioural activation and activity scheduling. Improve self-esteem / confidence building to develop stronger sense of personal agency. Promote positive beliefs about coping and resilience.
Longer term	Reclaim personhood and positive self-identity.	*“Getting me life back. Yeah and get back in work and get back to the person I used to be.”(P05).*	Develop stronger recognition of own values and hopes for the future. Re-establish connecting with previous achievements.
	Re-establishment / improvement of close relationships. Harnessing support and understanding of family.	*“And it would help others come to terms with the illness as well,* i.e. *your mum or your dad or your brother or your sisters who you live with or your partner.” (P01)*	Discuss possibility of information sharing with and/or involvement of family in therapy and longer-term suicide prevention plans.

#### Theme 1: A therapy that ‘works’

This theme reflected participants’ perceptions of context-specific internal and external influences and necessary features required for a therapy that would ‘work’ for suicidal psychiatric in-patients.

##### Past experiences shaped expectations

ᅟ

One-fifth of participants reported they had previously received psychological therapy, although only one participant had received therapy in a psychiatric ward setting. Some participants were perplexed about the different terms used to depict psychological therapy: “*I don’t know. What is psychological therapy? … I’ve done CBT?” (P09).*

CBT was the most commonly experienced therapy, although participants tended to speak of ‘counselling’ when referring to any type of psychotherapy. Discussions about experiences of therapy invariably gravitated to whether it had *‘worked’* or not.

Evaluations were judged according to perceptions of how successful therapy had been in addressing personally held goals, which for some, was eradication of particular symptoms:

*“I’ve had CBT, I’ve had lots and lots of therapies in [hospital]… when it comes to the severe depression, the suicidal feelings, the attempted suicides – they went away.”* *(P07)*

Dissatisfaction with psychological therapy resulted when it failed to meet prior expectations. Some participants were unprepared to openly discuss difficult and sensitive issues from their past, attributing subsequent worsening of their condition to such talk:

*“When I used to see the psychologist sometimes and started drinking heavier and heavier because I was having to talk a lot about me past and stuff… it was just all buried stuff.” (P02).*

For some, the therapist’s passive style was incompatible with their expectations: *“I’ve had counselling… it was drink-related but I didn’t find it helpful at all… all the guy did was just nod and it started to irritate me.” (P05).* An expectation of two-way communication was common and, where lacking, could precipitate collapse of the therapeutic relationship, *“It was me doing all the talking, it was him listening with no feedback and if I don’t get any feedback then I can’t work with anybody.” (P02).*

##### Suicidality-specific goals

Participants described some expectations or goals for therapy that were particularly related to addressing their suicidality (i.e., their suicidal thoughts or behaviours) which invariably prioritised relief from distress:

*“I’m willing to give anything a go to stop me feeling like this.” (P11).*

Another priority was discharge from hospital, yet participants understood that discharge was unlikely while still engaging in suicidal behaviour, making cessation of such behaviour important:

*“probably any idea or whatever to try and stop self-harming or doing overdoses … I just hope it [therapy] would help and then I can get discharged.” (P06).*

Participants perceived that suicide-focused therapy could offer a route to understanding and making sense of their suicidal crisis. Improved self-understanding and development of self-management strategies were seen as pivotal to avoid future suicidal behaviour representing strategies that could be used beyond hospitalisation:

“*Now, if you’re to sit me down and say, “Well, why do you want to take your life? What’s your thinking behind wanting to take your life? What’s causing you to think the way you do?” . . . looking at cognition, thinking styles, patterns, trying to challenge them.” (P07).*

*“help me to recognise when I’m going to be suicidal and perhaps be able to do something about it.” (P02).*

Suicide-focussed therapy was welcomed as participants’ recounted few opportunities to talk about being suicidal with ward staff. Talk was perceived as an essential vehicle for self-understanding with therapy being recognised as a potentially helpful intervention.

*“just talking about the suicidal thoughts… and why I’ve got them… talking about it would just help really ‘cos there’s no one to talk to about it, so it’d be best to just to have someone to talk to about the suicidal thoughts and what they’re about.” (P10).*

In expressing optimism towards the possibility of a suicide-focused psychotherapy, one participant alluded to the quintessential dilemma inherent in the suicidal crisis itself, of the ambivalence between wanting to die, and also wanting to be saved. This dilemma occurred at a time when help appeared inaccessible or unavailable suggesting that provision of a suicide-focused psychological therapy could address an important unmet need:

*“if I really wanted to kill myself I wouldn’t tell anybody I was going to do it I would go to a forest where no one would find me and hang myself from a tree… these people, who have had five failed suicide attempts, they are cries for help, they wanted to be found, because if you wanted to kill yourself, you’re not daft you know how to do it, wouldn’t you? It’s because they can’t get help, but now that your thing [suicide-focused therapy] will be implemented they won’t have to do that, think of all of the lives you’ll save.” (P12).*

##### Mechanisms: How suicide-focused therapy ‘works’

Participants perceived that therapy would work by fulfilling their need to talk about their suicidal thoughts and behaviours. They perceived this necessary to understand and make sense of their situation. The option of a suicide-focussed psychological therapy was attractive, as requests to talk with staff were frequently reported as unavailable leaving participants feeling isolated and frustrated when the only intervention offered was a pharmacological sedative.

*“in hospital they just ask you have you got them [suicidal thoughts] and if you have then they just try and give you some medication for it, they don’t talk about why you’ve got them…” (P10).*

Participants alluded to a belief that in-depth reflection and discussion of their innermost fears was necessary in order to overcome these, and that therapy would enable cathartic ventilation of worries and purposeful endeavour to problem-solve underlying issues.

*“Well, it’s to try to get you to open up isn’t it? ... And talk about things that are on your mind and your worries and all that. And I think the therapy part of it is to… help you to face your fears and your worries.” (P14).*

Therapy was seen as a route to recovery beyond hospitalisation during the suicidal crisis, offering individuals’ support to improve social confidence and other aspects of psychological wellbeing necessary for restoration of usual lifestyle roles despite the challenges of on-going problems. For example, recovery of lost personhood and meaningful occupation were important to participants:

*“Getting me life back. Yeah and get back in work and get back to the person I used to be.”(P05).*

*“building up my social skills a bit more and like talking to people in a group.” (P19).*

Participants also perceived that the benefits of therapy could extend to family members:

*“coming to terms with your illness is one part. And it would help others come to terms with the illness as well, i.e., your mum or your dad or your brother or your sisters who you live with or your partner.” (P01).*

Participants articulated clear expectations of the essential qualities necessary for an in-patient therapist, recognising the importance of a strong therapeutic relationship for successful therapy:

*“empathy, having the ability to climb into someone’s internal frame of reference*. *.*. *communicate on their wavelength. Being able to listen actively” (P07).*

#### Theme 2: Concerns about inpatient suicide-focused therapy

The second theme illustrated participants’ perceptions of contextual barriers for ward-based suicide-focused therapy specific to the therapeutic relationship and therapy process.

##### A secure therapeutic relationship

The primacy of a safe therapeutic relationship built on trust and confidentiality was universal among participants; *“If you don’t establish trust or the chemistry is wrong between the therapist and the client, it won’t work.” (P07). However,* there were concerns that trust could be particularly challenging for suicidal in-patients: *“I don’t know how you convince people to trust you when they’re finding it hard to trust people anyway?” (P14).*

Participants’ related past negative experiences of broken trust where disclosure of suicidal thoughts and covert suicidal acts to staff had resulted in undesirable consequences; *“I’ve opened up to people in the past and they’ve used it against me” (P14)*. However, mistrust was not global and fellow in-patients sometimes became trusted confidantes; *“last night I wanted to [ligature]*. *.*. *I can talk to my friend in here, not the staff” (P06).*

Similar concerns were held about confidentiality of therapy sessions: *“They [inpatients] can be very suspicious with therapies… they don’t trust the information they give will be confidential, even though they [therapists] say it will be…” (P07).* Tension existed between participants’ need to talk about suicide during therapy knowing that the therapist would have to inform ward staff should there be concerns of imminent risk of self-harm which could threaten willingness to engage in therapy and divulge suicidal thoughts:

*“If I told you I’m going to hang myself tonight, you’d have to tell the nurses, wouldn’t you?” (P13).*

Others were more comfortable with the therapist sharing risk information with ward staff, but would wish to impose restrictions and exercise control over the destiny of particularly personal information:

*“Well with me, it [sharing information] wouldn’t bother me. It just depends what they’re going to do with that information… How far is it allowed to go? ... In my case, there’d be one or two things where I wouldn’t be comfortable with staff knowing.” (P14).*

There were greater concerns about sharing information with relatives and some participants would refuse permission for the therapist to discuss matters with family:

*“The family, I think, would, that’s on a need-to-know basis. You don’t want to upset family members for no reason.”* (P11).

*“No, I wouldn’t want it shared with family and friends ‘cause it’s supposed to be a private session.”* (P10)*.*

##### Potential for harm

Emotional avoidance was evident with one participant alluding to a protective ‘barrier’ behind which intense and potentially overwhelming emotions were contained, being fearful of negative consequences if required to talk about sensitive matters; *“I’d be worried what will happen once that barrier’s been broke down to tell you the truth. Because I don’t know whether I’d start crying or get angry.” (P14).* Others feared that talking about being suicidal might make them more suicidal: *“… it might make me more suicidal…and if it’s prevalent, if it’s there and somebody’s reminding you of it, you’re more likely to do it” (P02).*

##### Ending therapy

There were concerns that abrupt ending of therapy could result in ‘unfinished business’ leaving individuals vulnerable and unsupported with suggestions for provision of post-discharge community therapy:

*“If you give therapy and it’s not completed, they may have issues, very sensitive, emotionally driven issues, that have been touched upon, which made it worse and it’s not been treated effectively. You can end up being worse off. Whereas, to give them the continuous care therapy in the community, if they do get discharged… that would be a very good thing” (P07).*

### A therapy that ‘works’: User-informed conceptual model of in-patient suicide-focused psychological therapy

Further analysis enabled creation of a framework linking user-informed acceptability prerequisites with tenets of suicide-focused psychological therapy [52] expressed as a tentative conceptual model (see Table 3). This offers information to assist future researchers and clinicians in delivering ‘psychologically-informed’ suicide prevention interventions and provides a benchmark for further testing and hypothesis development. A collective of participant’s expressed needs and valued outcomes have been aligned with temporal stages of suggested therapeutic techniques based on a cognitive behavioural suicide prevention therapy manual [52]. Flexibility and individualisation for particular clients is required. In the immediacy of suicidal crisis, individual’s needs for catharsis and relief of psychological distress by therapeutic talk within a safe, secure and trusting therapeutic relationship are prioritised. Following this, the need for self-understanding to enable proactive self-care and avert future suicidal behaviour is suggested. Quality of life and functional outcomes were also seen as important to support and sustain maintenance of improved psychological health.

## Discussion

### Main findings

This is the first study to investigate suicidal in-patients views and expectations of suicide-focused psychological therapy and offers important insights of the ‘real-world’ contextual needs and challenges around provision of psychological therapy for suicidal in-patients.

### Incompatibility of service objectives and in-patient needs

Dominating the findings was in-patients’ needs for therapeutic talk to process, make sense of, and address their suicidality which does not accord with prevailing models of psychiatric treatment for suicidal in-patients, which prioritize physical safety by observation and containment [58]. Insufficient opportunity to explore the emotional impact of being suicidal and to find solutions to the problems that precipitated admission creates the potential for reinforcement and entrenchment of the in-patients’ status quo. Despite the existence of scientific knowledge of the modifiable psychological factors which have been shown to be associated with suicidality (i.e., hopelessness, defeat and entrapment [59]), we found that suicidal inpatient participants’ reported no opportunities to receive any psychological treatment. Such lack of attention to the psychological architecture of suicidality along with the associated negative experiences of in-patient treatment may be contributory factors to the persistently high ‘revolving-door’ readmission rates and post-discharge escalations of suicide rates [4, 48, 60].

Participants described how attempts to discuss suicidal thoughts with staff were thwarted by rebuffs or offers of sedative medication to the detriment of addressing their needs for therapeutic talk. Whilst sedation offers temporary relief from intense emotional distress, it is not an appropriate substitute for substantive treatment of suicidality, and used in this manner may impose a state of emotional avoidance likely to perpetuate psychological morbidity [61–64]. This exposes a major conflict between the aims and priorities of staff and in-patients. In reality, staff have limited access to nonpharmacological interventions and psychiatric wards are busy, unpredictable and impose vast personal demands on staff caring for suicidal in-patients [5, 46, 58]. A recent study of ward staff experiences of working with suicidal in-patients identified a culture where risk aversive avoidance of suicide-talk dominated their practice highlighting the need for staff training [65]. Indeed the results from this study of suicidal inpatients’ experiences confirm this and demonstrate the impasse between the needs and fears of suicidal in-patients and those of ward staff adding further support of the need for ward staff training.

### Barriers to suicide-focussed therapy

#### Past negative experiences

Some participants held negative views of psychological therapy based on past unsatisfactory experiences, for example, discussion of sensitive matters during therapy was perceived to have worsened their condition. Past experiences are important as they impact on expectations of future therapy [28, 66] and where negative, are likely to adversely affect desire and willingness to engage in further therapy. The notion of ‘negative therapeutic reaction’ is recognised within psychotherapy, commonly presenting during initial therapy sessions as a transient sequel to discussion of painful and deeply suppressed issues [67]. Therefore, to avoid disengagement or withdrawal from therapy, it is important to inform recipients of the potential for a transient worsening of their condition and discuss how this would be managed. Some participants perceived therapist passivity as disengaging, indicating the need to discuss role expectations with potential clients as pre-therapy ‘*role induction*’ is strongly associated with reduced attrition and better clinical outcomes [68].

#### Fears of increased suicidality

Participants viewed relief of distress as the prime goal of therapy, yet some feared that suicide-focussed therapy might increase distress or even trigger suicidal ideation representing a potential barrier to accepting such treatment. Willingness to talk about suicide is essential for suicide-focussed psychological therapy, and indeed, has been shown to be a key aspect of effective psychological treatments for suicide [59]. However, this presented particular challenges concerning trust and confidentiality for psychiatric in-patients, as many are involuntarily detained [69]. Although participants understood the therapist’s responsibility to break confidentiality by informing ward staff of heightened suicide risk [70], this did present a potential barrier to uptake and engagement in suicide-focused therapy. A paradox existed whereby inpatients wanted to talk about their distress and suicidal thoughts, yet recognised discharge as unlikely should staff become aware of their persistent suicidal ideation. This quandary may have led some in-patients to feign cessation of suicidal thoughts in order to gain discharge [71] and may also be implicated within the escalation of suicide rates during the immediate post-discharge period [4].

### Strengths

The contextual challenges of conducting research in psychiatric wards are extensive [72]. However, our results offer particular insights to assist others wishing to implement psychological treatments in this setting. Participants raised many context-specific concerns which are important to consider as contextual influences within treatment settings impact on the therapeutic alliance and ultimately on the treatment effect [73, 74].

Our study researched the expectations of suicidal in-patients as part of the development of a suicide-focussed psychological intervention. Our participants were not involved in the clinical trial of this intervention but their status mirrored the eligibility requirements of trial participants thereby enabling them to represent a ‘by-proxy potential participant’ identity. This may help to overcome a recognised limitation with traditional approaches of participant evaluations *following* receipt of therapy which frequently report high levels of positive affirmations suggestive of social desirability bias [66]. We therefore suggest an important role for investigation of potential recipients’ views and expectations as a method to enhance the ecological validity of novel psychological treatments. Patient acceptability of treatments aimed at suicidal in-patients is particularly important as their admission presents a critical opportunity to intervene and save life [10] which for some, (given that 43% of inpatient suicides occur in the month following discharge, and of these, 47% had died in the first week prior to receiving any community follow-up [75]), may represent our last opportunity.

Further, this study demonstrates the unique role of qualitative approaches in ‘giving voice’ to highly sensitive, often covert, personal views of vulnerable populations [76]. Our sample comprised suicidal psychiatric inpatients from whose rich accounts a unique data-driven user-informed model for suicide-focussed psychological therapy was devised. Our model, unlike others that have relied on secondary documentary sources [77], represents contemporary ‘real-world’ verbatim accounts directly obtained from participants’ whose precise clinical and contextual situation mirrored that of individuals who would be eligible for future ward-based suicide-focused therapy. As such this affords a high level of ecological validity.

### Limitations

It is possible that individuals who volunteered to participate may have been more comfortable and interested in talking about suicide and we may not have recruited people who would be less likely to engage in a suicide-focused talking therapy. By accessing individuals outside from the therapy trial this bias is likely to be minimised. Nevertheless it is possible that findings relating to concerns about suicide-focused therapy are underestimated and the need to address these concerns may be lacking from the user-informed model of suicide-focussed psychological therapy which derived from the analysis. As only one-fifth of participants had any prior experience of psychological therapy their views should be interpreted cautiously. A further limitation is that our sample was drawn from one (albeit large) mental health trust and further study with larger samples of broader sociodemographic variability including more balanced gender composition is indicated.

### Recommendation for research and clinical practice

We have demonstrated that the suicidal in-patient participants in this study would welcome ward-based suicide-focussed psychological therapy as an additional treatment choice as recommended in UK statutory guidelines [16]. Further research with a sufficiently powered sample size is required to determine efficacy and to further advance intervention development regarding effectiveness with all stakeholders. Participant generated constructs within our user-informed model of suicide-focused therapy (See Table 3) offers a basis for further research to develop a user-defined outcome measure of recovery from suicidal episodes. More generally, further research to uncover and generate workable solutions for the precise organisational and ward level cultural dynamics that perpetuate the dissonance of needs and views of suicidal inpatients and the ward staff who look after them is required.

Our approach of formal research investigation to exploring the views, expectations and priorities of individuals who would fulfil eligibility criteria for the intervention being developed may be a useful pre-randomised clinical trial phase to extend to other new treatments and populations. However, further research with longitudinal correlational design would be required to determine how this approach impacts on research participant recruitment, retention and definitive trial clinical outcomes. Recipient understanding of the nature of any treatment is essential for ethical research [70] and our findings confirm the importance of providing potential participants’ with information of the key requirements of complex therapy, especially if willingness to discuss distressing topics is necessary. Table 4 contains a body of user-generated issues constituting potential avoidable barriers and associated recommendations as guidance for clinicians / research therapists pursuing the practice of suicide-focused psychological therapy.

**Table 4:** Recommendations for research and practice of suicide-focused psychological therapy

In-patients’ views	Implications for suicide-focused therapy	Recommendations for therapist
Past negative experiences of therapy	Unsatisfactory previous experience of therapy may prevent uptake of suicide-focused therapy. May lead to avoidable dissatisfaction and attrition if therapist not aware of clients’ expectations and preferences.	Enquire about and consider the impact of any past experiences of therapy. Provide clear information about the nature and demands of suicide-focused therapy and the potential for negative therapeutic reaction.
Confusion / lack of understanding of aims and functions of psychological therapy	Potential for disappointment if client’s expectations of therapy cannot be met. Need to identify and manage realistic expectations. Need for active client engagement during and in-between sessions.	Discuss and mutually agree expectations including client’s expectations of own and therapist’s role, and therapist’s expectations of client’s role.
Concerns about trust and confidentiality of information disclosed in therapy	Lack of trust and confidence in confidentiality may impact on continued uptake and engagement. Demonstration of openness and transparency by therapist explaining own responsibility for breaking confidentiality if concerned for client’s safety may serve to build trust. Therapist must inform ward team if actual or risk of harm to client or others disclosed in therapy. Need to clarify client’s wishes of who non-risk information may or may not be shared with.	Allow time for trust to develop recognising the particular challenges for inpatients who may be involuntarily detained. Demonstrate consistent reliable behaviour Discuss limits of confidentiality with client. Discuss when and how any disclosures of actual or risk of harm to self or others would be managed respecting clients’ preferences where possible. Agree what and with whom other information may be shared with (e.g., ward staff, family).
Fear or unwillingness to talk about suicide	Willingness to discuss suicide is essential for suicide-focused therapy. Covert fears of potential for harm may impede engagement in therapy.	Important to give full information about the need to talk about suicide to enable informed consent. Proactive discussion to elicit any client fears about perceived dangers of talking about suicide. Provide reassurance that client will retain control of the depth and pace of therapy.
Concerns about the ending of therapy	Anxiety about possibility of abrupt ending of sessions may affect ability to engage in therapy.	Involve client in discussions about preferences for ending of therapy. Offer ways of gradual spacing of session intervals, follow-up or booster sessions.

## Conclusion

Hospitalisation of suicidal individuals offers a critical opportunity to engage inpatients in effective treatment and preserve life. Provision of ward-based suicide-focused psychological therapy was cautiously welcomed by suicidal in-patients whose expressed needs, concerns and priorities have been presented and collated into a user-informed model of the key components of suicide-focussed therapy. Patients comprise the largest and potentially most influential stakeholder group in terms of ultimate uptake of health interventions and opportunities to enhance treatment acceptability by understanding their ‘real-world’ context offer great promise yet remain underexploited [78]. Notwithstanding the need for further research, we cautiously suggest that pre-clinical trial investigation of research participants’ views of novel treatments may positively contribute to the future development of better quality treatments likely to improve participant / patient uptake whilst also optimising efficient use of public funding.

## Abbreviations

CBT: Cognitive Behavioural therapy
DBT: Dialectical Behavioural Therapy
NHS: National Health Service
NICE: National Institute for Health and Care Excellence
